# SU5416 does not attenuate early RV angiogenesis in the murine chronic hypoxia PH model

**DOI:** 10.1186/s12931-019-1079-x

**Published:** 2019-06-17

**Authors:** Grace L. Peloquin, Laura Johnston, Mahendra Damarla, Rachel L. Damico, Paul M. Hassoun, Todd M. Kolb

**Affiliations:** 1 Division of Pulmonary, Critical Care, and Sleep Medicine, Department of Medicine, NYU Langone Health, New York, NY USA; 2 Division of Pulmonary and Critical Care Medicine, Department of Medicine, Johns Hopkins University School of Medicine, 1830 E. Monument Street, 5th Floor, Baltimore, MD 21205 USA

**Keywords:** Right ventricle, Angiogenesis, Pulmonary hypertension, Cardiac endothelial cell, VEGFR-2

## Abstract

**Background:**

Right ventricular (RV) angiogenesis has been associated with adaptive myocardial remodeling in pulmonary hypertension (PH), though molecular regulators are poorly defined. Endothelial cell VEGFR-2 is considered a “master regulator” of angiogenesis in other models, and the small molecule VEGF receptor tyrosine kinase inhibitor SU5416 is commonly used to generate PH in rodents. We hypothesized that SU5416, through direct effects on cardiac endothelial cell VEGFR-2, would attenuate RV angiogenesis in a murine model of PH.

**Methods:**

C57 BL/6 mice were exposed to chronic hypoxia (CH-PH) to generate PH and stimulate RV angiogenesis. SU5416 (20 mg/kg) or vehicle were administered at the start of the CH exposure, and weekly thereafter. Angiogenesis was measured after one week of CH-PH using a combination of unbiased stereological measurements and flow cytometry-based quantification of myocardial endothelial cell proliferation. In complementary experiments, primary cardiac endothelial cells from C57 BL/6 mice were exposed to recombinant VEGF (50 ng/mL) or grown on Matrigel in the presence of SU5416 (5 μM) or vehicle.

**Result:**

SU5416 directly inhibited VEGF-mediated ERK phosphorylation, cell proliferation, and *Kdr* transcription, but not Matrigel tube formation in primary murine cardiac endothelial cells in vitro. SU5416 did not inhibit CH-PH induced RV angiogenesis, endothelial cell proliferation, or RV hypertrophy in vivo, despite significantly altering the expression profile of genes involved in angiogenesis.

**Conclusions:**

These findings demonstrate that SU5416 directly inhibited VEGF-induced proliferation of murine cardiac endothelial cells but does not attenuate CH-PH induced RV angiogenesis or myocardial remodeling in vivo.

**Electronic supplementary material:**

The online version of this article (10.1186/s12931-019-1079-x) contains supplementary material, which is available to authorized users.

## Background

Limited understanding of mechanisms driving right ventricular (RV) adaptation in pulmonary hypertension (PH) prevents the development of much needed therapies targeted to RV failure. Considerable attention has recently been focused on the role of RV angiogenesis in preventing the transition to RV failure in PH. Pre-clinical PH models have generally demonstrated associations between RV angiogenesis and adaptation (preserved cardiac function) and RV capillary rarefaction with RV failure [1–7]. Recently, unbiased stereological methods have demonstrated that attenuated RV angiogenesis accounts for capillary rarefaction in the SU5416/Hypoxia (SuHx) rat model of severe PH [7], similar to observations in a small group of human PAH samples [8]. The critical molecular regulators governing adequacy of RV angiogenesis are unknown.

Vascular endothelial growth factor receptor-2 (VEGFR-2) is a receptor tyrosine kinase, expressed primarily on endothelial cells (EC) [9, 10], and generally regarded as a “master regulator” of angiogenesis due to its critical role in EC differentiation, proliferation, migration, and formation of the vascular tube (reviewed in [11]). The small molecule tyrosine kinase inhibitor SU5416 is a 3-substituted indolin-2-one compound with relatively high specificity for VEGFR-2 and VEGFR-1 [12, 13], used extensively in animal models of PH [14], primarily due to effects on pulmonary vascular endothelial cell apoptosis and proliferation. Little is known about the direct effects of SU5416 on coronary ECs in these models. Most studies measuring the effect of SU5416 on EC function have focused on human umbilical vein endothelial cells (HUVECs), though a single study has shown that the compound can inhibit VEGF-dependent proliferation and sprouting, but not tube or spheroid formation, of an immortalized cardiac microvascular endothelial cell line in vitro [15]. However, the effects of SU5416 on primary cardiac EC function in PH has not been explored.

We previously reported on a murine model of chronic hypoxia-PH (CH-PH) induced RV angiogenesis, which was associated with protection from myocardial hypoxia and preserved cardiac function [6]. Some investigators have previously demonstrated exacerbation of murine CH-PH with SU5416, postulating that prolonged depression of RV function was related to direct effects of SU5416 on the RV myocardium [16]. Effects of SU5416 on RV angiogenesis in the murine CH-PH model have not been assessed. We hypothesized that SU5416, through direct effects on cardiac ECs, would attenuate RV angiogenesis in this model. We show here that VEGF-dependent receptor activation and EC proliferation, but not tube formation are attenuated by SU5416 in primary cardiac endothelial cells in vitro. However, SU5416 administration does not inhibit early RV angiogenesis in the CH-PH model, despite altering global RV angiogenic gene expression.

## Methods

### Murine cardiac EC culture

Primary cardiac endothelial cells isolated from C57 BL/6 mice (MCEC; Cell Biologics, cat. #C57–6024, Chicago, IL, USA) were cultured on gelatin-coated plates and maintained in complete EC media supplemented with vascular endothelial growth factor (VEGF), endothelial growth supplement, heparin, epidermal growth factor, hydrocortisone, L-glutamine, antibiotic-antimycotic solution, and 5% fetal bovine serum (FBS), according to the manufacturer’s recommendations. EC phenotype was independently confirmed by measurement of acetylated-LDL uptake (5 μg/mL for 4 h; Additional file 1 :Figure S1). All experiments were performed between passage 3–7 in Dulbecco’s Modified Eagle’s medium (DMEM; Thermo Fisher Scientific; Waltham, MA), supplemented with minimal (2%) fetal bovine serum. MCEC were maintained in treatment media for 16 h prior to incubation with SU5416 (5 μM) or vehicle (DMSO) for two hours, then treatment with recombinant VEGF-A_165_ (50 ng/mL; R&D Systems, Minneapolis, MN, USA) or vehicle (0.1% BSA in PBS). Cell lysates were prepared in Laemmli buffer (Western blot) or Trizol Reagent (quantitative PCR). Proliferation was measured by counting cells with an automated handheld counter (Millipore Sigma; Burlington, MA) after 24–48 h of exposure to VEGF or vehicle. EC tube formation on Matrigel (Millipore Sigma) was measured after 18 h in complete EC media, with or without SU5416. Five images were obtained from each experiment, and total branching and number of nodes were quantified using Angiogenesis Analyzer Plug-in for Image J [17].

### Murine chronic hypoxia-PH model

Adult male C57 BL/6 mice (Jackson Laboratories, Bar Harbor, ME, USA), aged 8–10 weeks, were exposed to normobaric hypoxia (10%) for up to three weeks. Mice received a subcutaneous injection of SU5416 (20 mg/kg in 0.5% carboxymethylcellulose sodium, 0.9% sodium chloride, 0.4% polysorbate 80, 0.9% benzyl alcohol) or vehicle at the beginning of the CH exposure, and weekly thereafter (for longer experiments). CH-PH mice were maintained in a ventilated plexiglass chamber, and O_2_ concentration was maintained at 10% via nitrogen administration, controlled by a programmable O_2_ controller (Pro-Ox 110; Biospherix, Lacona, NY, USA). Ambient CO_2_ was controlled within the chamber using soda-lime, and the chamber was opened twice weekly for bedding changes. Chow and water were available ad libitum*.* Control mice were similarly maintained under ambient conditions adjacent to the plexiglass chamber.

After 3 weeks of CH-PH, mice were anesthetized with intraperitoneal pentobarbital (60 mg/kg) and mechanically ventilated with the following parameters: tidal volume = 10 mL/kg; rate = 160 breaths/min. Core temperature was maintained at 37 °C (±0.4°) using a heating pad controlled by a proportional-integral-derivative temperature control unit (Doccol, Sharon, MA, USA). The apex of the heart was exposed, and a1.2F catheter (Transonic Systems Inc., Ithaca, NY, USA) was inserted into the RV for continuous pressure measurement using a PowerLab data acquisition system (ADInstruments, Inc., Colorado Springs, CO, USA). Continuous pressure data were analyzed off-line using LabChart 7 software (ADInstruments, Inc.). Mice were then exsanguinated under anesthesia, and hearts were removed for measurement of RV hypertrophy, quantified as the mass ratio of RV free wall to LV and septum (Fulton Index) or body mass.

In some experiments, RV free wall and LV/septal specimens were flash-frozen immediately after euthanasia for molecular analyses after shorter CH-PH exposures. Tissue homogenates were prepared in RIPA Lysis and Extraction Buffer (protein) or Trizol Reagent (RNA; Thermo Fisher Scientific, Inc.) using the Bullet Blender Homogenizer cell disrupter (Next Advance, Inc., Averill Park, NY, USA). All animal protocols were performed in compliance with approved protocols by the Johns Hopkins IACUC.

### Western blot

Cell lysates and tissue protein homogenates were separated by SDS-PAGE and transferred to nitrocellulose membranes using standard methods [18, 19]. Western blot was performed using commercially available antibodies (Cell Signaling Technology; Danvers, MA) directed against VEGFR-2 (cat. #9098), phospho-VEGFR-2 (Tyr1175; cat. #2478), ERK (cat. #4695), phospho-ERK (Thr202/Tyr204; cat. #9101), GAPDH (cat. #3683), Actin (cat. #5125), and hsp90 (cat. #4874). All antibodies were diluted 1:1000 in Tris-buffered saline containing 0.1% Tween-20 (TBST) and 2.5% bovine serum albumin and incubated with the membrane overnight at 4 °C with agitation. Horseradish peroxidase (HRP)-labeled anti-rabbit antibody (Cell Signaling Technology cat. #7074) was diluted 1:10,000 in TBST with 5% non-fat dry milk (BioRad; Hercules, CA) and incubated for 1 h at room temperature. HRP was detected using Supersignal substrate (Thermo Fisher Scientific), and chemiluminescent signal was transferred to film and scanned for quantification using Image J software [17].

### Quantitative PCR and Array analysis

Total RNA homogenates from cells and tissue homogenates were extracted with chloroform, precipitated with isopropanol, and washed with ethanol prior to purification with commercially available columns (QIAgen, Germantown, MD, USA). Purified RNA was used to generate cDNA with the RT2 First Strand system (QIAgen). Quantitative PCR was performed using commercially available primers specific for mouse *Kdr* and *Gapdh* (QIAgen) and the iTaq SYBR green system (BioRad). Cq values were generated using freely available software [20], and RNA expression was compared between groups using the ∆∆Cq method [21]. In some experiments, total RNA isolated from flash-frozen RV was used to compare global expression profiles of angiogenesis-related genes using a commercially available microarray (RT2 Profiler, Qiagen). Following RNA purification and cDNA generation, quantitative PCR array analysis was performed using the Stratagene Mx3005P PCR system (Agilent Technologies; Santa Clara, CA). Data analysis was performed off-line using the ∆∆Cq method, with changes in expression referenced to an unbiased gene selected by analysis of the entire expression array (*Egf*). Microarray analyses were performed using freely available online software (Qiagen).

### Stereology: quantification of RV capillary length, surface, and volume

Fully anesthetized mice were treated with intravenous heparin (50 IU), and the heart was arrested in diastole by intravenous injection of saturated potassium chloride prior to exsanguination. Hearts were removed and preserved by immersion fixation in 10% formalin. Following removal of the atria and great vessels, the RV free wall was dissected from the septum and LV, and both ventricles were weighed. RV mass was converted to a reference volume by dividing by the density of muscle tissue (1.06 g/cm^3^), as previously reported [22–24]. The RV was cut into 8–10 blocks of approximately 2 × 2 mm. Four blocks were chosen by systematic uniform random sampling; these were pre-embedded in spherical agarose molds to generate isotropic uniform random (IUR) sections in paraffin according to the isector method [25]. IUR sections on glass slides were de-paraffinized using xylene and a graded ethanol series and stained with fluorophore-conjugated probes specific for EC glycoproteins (Isolectin GS-IB_4_ from *Griffonia simplicifolia* AlexaFluor-568 conjugate, diluted 1:100 in PBS; Thermo Fisher Scientific cat. #I21412) and myocyte plasma membrane glycoproteins (wheat germ agglutinin, AlexaFluor-488 conjugate, diluted 1:200 in PBS; Thermo Fisher Scientific), as previously described [26]. Digital images were obtained from ten 60X fields per section, and interactions of capillaries (< 10 μm diameter) and myocytes with stereological probes for estimating capillary length (plane), capillary surface area (line), and capillary/myocyte volume (points) were quantified in a blinded manner using freely available software [27]. Interactions were used to estimate length, surface, and volume density, as previously reported [6]. Estimated densities are shown in Additional file 3: Figure S3. Parameter densities were then converted to total RV capillary length, capillary surface area, and capillary/myocyte volume by multiplying by the reference (RV) volume for each animal. Average cross-sectional area supplied per capillary was estimated by dividing the reference volume (cm^3^) by the total capillary length (cm). Radius of RV tissue served per capillary was then estimated by dividing this area by π and taking the square root of the result.

### Immunohistochemistry

In some experiments, formalin-fixed, paraffin-embedded RV sections were stained for VEGFR-2 expression or proliferating cell nuclear antigen (PCNA) following antigen retrieval with citrate-EDTA buffer [28]. Non-specific binding was blocked by incubation with 5% normal goat serum, and endogenous biotin, biotin receptors, and avidin binding sites were blocked using the Avidin/Biotin Blocking Kit (Vector Laboratories; Burlingame, CA). Primary antibodies (Cell Signaling Technology cat. #9698, 1:200; #13110, 1:500) were diluted in TBST containing 1% normal goat serum and applied to sections for 40 min at room temperature. Primary antibody was detected using an avidin-biotin system (Vector Laboratories;), and HRP activity was visualized with 3,3′-diaminobenzidine or NovaRed substrates (Vector Laboratories).

### Flow cytometry

Mice were euthanized under anesthesia and hearts removed for preparation of single-cell suspensions. The RV free wall was dissected from the LV and septum. Both fractions were minced and incubated in RPMI media containing 0.25% collagenase I (Worthington Biochemical Corporation, Lakewood, NJ, USA) and 0.05% DNase I (Sigma-Aldrich, Saint Louis, MO, USA) for 45 min at 37 °C. Suspensions were triturated through a 14-g needle and filtered through a 70 μm mesh nylon strainer to remove clumps. Erythrocytes were removed by incubation with ACK lysis buffer, and cells were washed and re-suspended in PBS with 1% BSA. Single-cell suspensions were stained for 30 min (4 °C) with the LIVE/DEAD Fixable Blue Cell Stain Kit (cat.#L23105, Thermo Fisher Scientific), and non-specific labeling of mouse Fc receptors was blocked by incubation with anti-mouse CD16/CD32 antibody (cat. #553142, BD Pharmingen; San Jose, CA; 1:100) for 10 min at 4 °C. Surface antigens were labeled by incubation with anti-mouse CD31 (cat. #102422, Biolegend, Inc.; San Diego, CA; 1:167 in PBS/1% BSA) and anti-mouse CD45.2 (cat. #109823, Biolegend, Inc.; 1:500 in PBS/1% BSA) monoclonal antibodies for 20 min in the dark at 4 °C prior to fixation (30 min at 4 °C) and permeabilization (Foxp3/Transcription Factor Staining Buffer Set; eBioscience, Inc., San Diego, CA). Fixed and permeabilized single-cell suspensions were then incubated for 30 min (4 °C) with an anti-human Ki-67 antibody (recognizes mouse Ki-67; cat. #51-36,525X, BD Pharmingen; 1:200 in permeabilization buffer). The staining protocol was replicated with matching isotype control antibodies for anti-mouse CD31 (cat. #400527, Biolegend, Inc.) and anti-human Ki67 (cat. #51-35,405X, BD Pharmingen) at the same dilutions. Stained RV and LV cell suspension were then subjected to flow cytometry using a FACSAria cell sorter and analyzer (BD Biosciences). Compensation was performed using UltraComp eBeads (eBioscience, Inc.) for each antibody, single-stained cells for LIVE/DEAD, and unstained cells for background autofluorescence. Data were analyzed using FlowJo software (v. 7.6.5; Ashland, OR, USA), as depicted in Fig. 4. The percentage of live, CD31+/CD45.2- cells staining positively for Ki-67 (% Ki-67+) was used as a measure of EC proliferation.

### Statistical analysis

All data are expressed as mean ± standard error of the mean. Comparison of means between two groups was made using an unpaired student’s t-test, with Welch’s correction in the setting of unequal variances, as appropriate. Comparison of means between control groups, CH-exposed groups, and SuHx-treated groups at multiple time points was made using one-way ANOVA, with post hoc testing corrected for multiple comparisons using Dunnett’s test. A two-tailed *p* < 0.05 was considered statistically significant in all experiments. Statistical analyses were performed using GraphPad Prism software (v 7.0; GraphPad Software, Inc., La Jolla, CA, USA).

## Results

### SU5416 inhibits VEGFR-2 signaling and proliferation of primary mouse cardiac endothelial cells in vitro

**Fig. 1:**
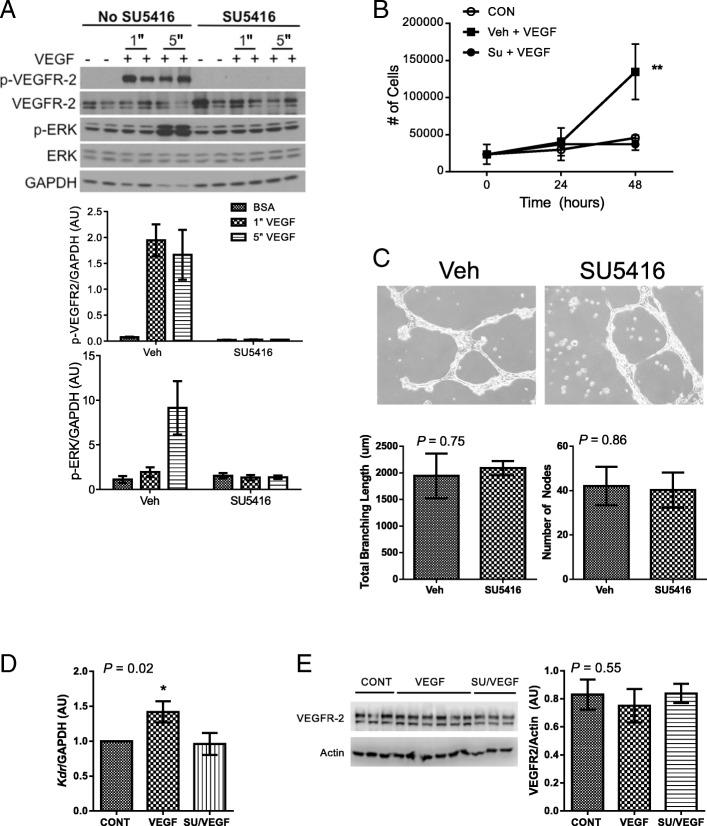
Effects of SU5416 on primary MCEC in vitro. (**a**) When exposed to recombinant VEGF (50 ng/mL) in vitro, signaling through VEGFR-2 is rapidly activated, as demonstrated by phosphorylation of VEGFR-2 (Y1175) and ERK. Pre-treatment with 5 μM SU5416 completely inhibits VEGFR-2 Y1175 phosphorylation and subsequent ERK phosphorylation. Graphs show densitometry from *n* = 4/group. "denotes time in minutes. (**b**) Pre-treatment with 5 μM SU5416 attenuates recombinant VEGF-induced cell proliferation (*n* = 3/group). (**c**) Tube formation on Matrigel is not inhibited by SU5416 (5 μM). Representative images are shown, as are quantitative estimates of total branching length and number of nodes calculated from n = 3 experiments/group. (**d**) SU5416 pre-treatment (5 μM) inhibits recombinant VEGF mediated increase in *Kdr* (VEGFR-2) transcription (*n* = 4/group) 6 h following stimulation. (E) VEGFR-2 protein levels are not increased 18 h after stimulation with recombinant VEGF, and this effect is not modified by SU5416 pre-treatment (5 μM). All error bars represent SEM. * denotes *P* < 0.05; ** denotes *P* < 0.01. Additional *P*-values are for Student’s t-test (C) or one-way ANOVA (**d**, **e**)

VEGFR-2 is the primary mediator of VEGF-induced EC signaling and pro-angiogenic phenotype. Stimulation of VEGFR-2 by VEGF promotes EC proliferation via Y1175 phosphorylation-dependent activation of extracellular signal-regulated kinase (ERK) signaling pathway [11]. Consistent with these observations in other EC types, administration of exogenous recombinant VEGF-165 to MCEC caused rapid VEGFR-2 activation, with phosphorylation of the receptor at the critical Y1175 site within 1 min, and subsequent phosphorylation of ERK within 5 min (Fig. 1a). Pre-treatment with SU5416 completely inhibited VEGF-dependent phosphorylation of MCEC VEGFR-2 and subsequent ERK phosphorylation. Recombinant VEGF also promoted MCEC proliferation, and this was completely suppressed by SU5416 pre-treatment (Fig. 1b).

Angiogenesis is a complex process, and later stages require additional changes in EC phenotype, including migration and differentiation. These events are also regulated by signaling pathways downstream of activated VEGFR-2 [11] . Tube formation on Matrigel is a well characterized in vitro assay reflecting these later stages of angiogenesis [29]. In contrast to the inhibitory effects of SU5416 on MCEC proliferation observed above, the compound did not inhibit MCEC tube formation on Matrigel (Fig. 1c).

VEGF-stimulation also promotes transcriptional up-regulation of *Kdr* (VEGFR-2) in other EC models, an effect that is dependent on VEGFR-2 tyrosine kinase activity [30]. Given the critical role of VEGFR-2 activation in EC signaling, increased receptor expression might promote angiogenesis. Stimulation of MCEC with recombinant VEGF-165 was associated with increased *Kdr* transcription after 6 h (Fig. 1d), as previously observed. VEGF-stimulated *Kdr* transcriptional up-regulation was completely suppressed by SU5416 pre-treatment. However, VEGF-induced increases in *Kdr* transcription were not associated with subsequent differences in MCEC VEGFR-2 protein levels 18 h after addition of recombinant VEGF (Fig. 1e), highlighting the complexity of VEGFR-2 regulation [31].

### SU5416 does not inhibit RV angiogenesis in mice exposed to CH-PH

We previously demonstrated that chronic hypoxia-induced PH in C57 BL/6 mice caused RV-specific angiogenesis, characterized by RV-specific increases in total capillary length and endothelial cell proliferation [6]. Molecular regulators of CH-PH induced RV angiogenesis in this model are unknown. Given the inhibitory effects of SU5416 on MCEC VEGFR-2 activation, ERK phosphorylation, proliferation, and receptor transcription observed in vitro, we hypothesized that SU5416 might attenuate CH-PH induced RV angiogenesis through similar inhibition of RV EC VEGFR-2 activity in vivo.

**Fig. 2:**
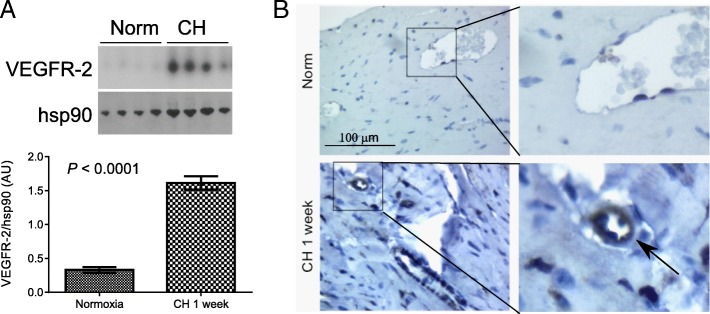
Right ventricular VEGFR-2 expression in CH-PH. VEGFR-2 expression is increased in the RV of mice exposed to CH-PH for 1 week, as demonstrated by western blot (**a**). Graphs denote relative expression (normalized to RV hsp90 expression) of n = 4 animals/group. Immunohistochemistry (**b**) identifies perivascular staining in RV sections from mice exposed to CH-PH, consistent with an endothelial cell source for observed increases in tissue VEGFR-2. Error bars represent SEM. *P*-value is for Student’s t-test

VEGFR-2 expression is generally limited to active, proliferative EC populations in adult tissues [9, 10]. We initially measured VEGFR-2 expression in RV, LV, and lung tissue homogenates from mice exposed to CH-PH for one week, a time point we have previously associated with RV-specific increases in total capillary length and EC proliferation in the CH-PH model [6]. We observed a marked, RV-specific increase in VEGFR-2 protein expression after 1 week of CH-PH (Fig. 2a). There was no increase in LV VEGFR-2 expression, and lung VEGFR-2 decreased at this time point (Additional file 2 :Figure S2). Immunohistochemical staining confirmed a perivascular distribution RV VEGFR-2 protein expression, consistent with an EC source (Fig. 2b).

**Fig. 3:**
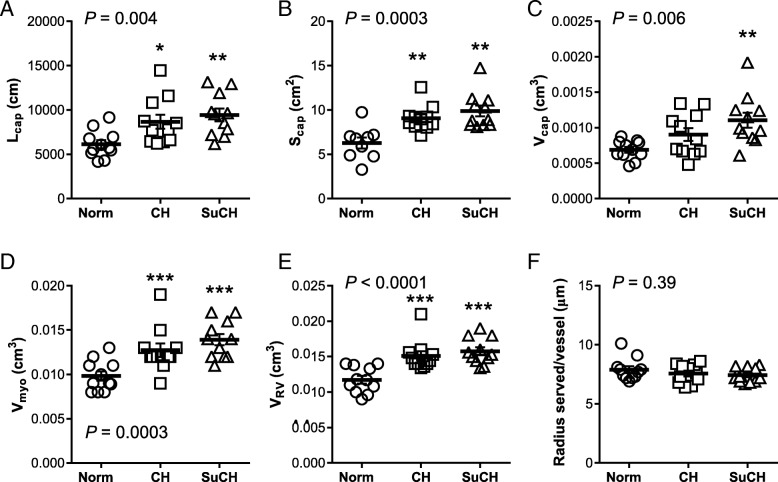
Effects of SU5416 on RV angiogenesis in the CH-PH model. After 1 week of exposure to CH-PH, SU5416 had no effect on observed increases in total RV capillary length (**a**; L_cap_), surface area (**b**; S_cap_), or volume (**c**; V_cap_), as estimated by unbiased stereological methods. Similarly, there was no effect of SU5416 on CH-PH induced increases in RV myocyte volume (**d**; V_myo_) or RV volume (**e**; V_RV_), and the estimated radius of tissue served/vessel did not change in either group (**f**). *P*-values are for one-way ANOVA. * denotes *P* < 0.05, ** denotes *P* < 0.01, and *** denotes *P* < 0.001 in comparison to normoxic controls (Norm). Data from all animals are shown; *n* = 11 animals/group

**Fig. 4:**
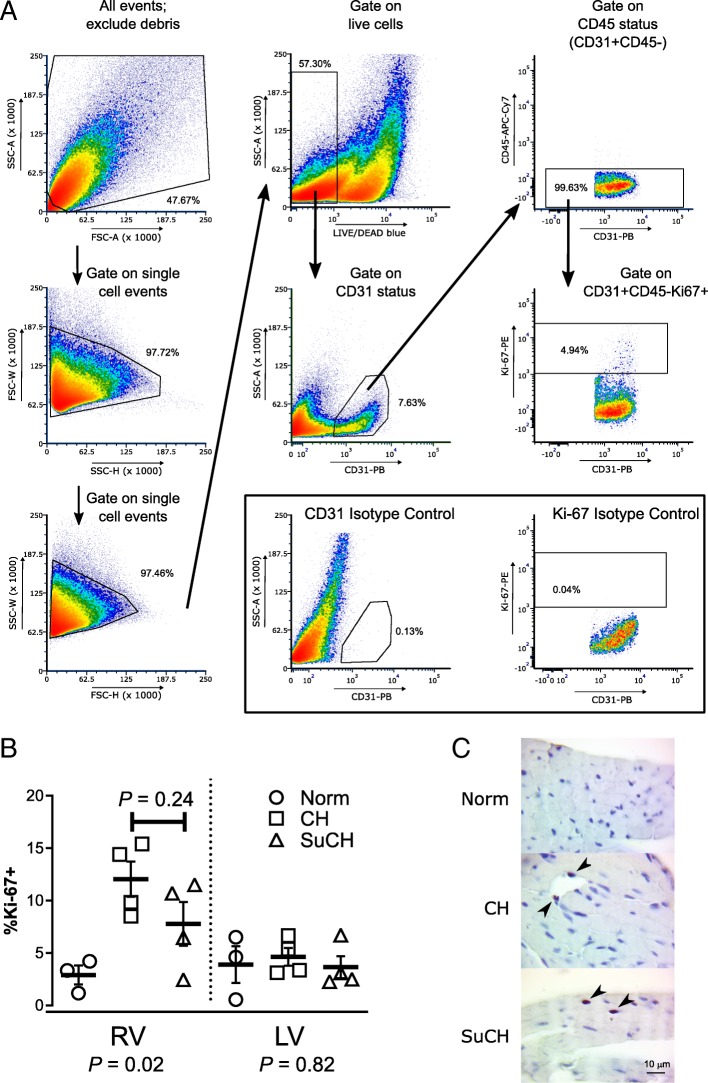
Effects of SU5416 on RV endothelial cell proliferation in the CH-PH model. The gating strategy used for analysis of flow data is shown in (**a**). Forward (FCS) and side scatter (SSC) gating by area (A), height (H), and width (W) was used to exclude debris and non-single cell events. Live cells were selected by gating on those excluding LIVE/DEAD blue stain. Live cells that stained positively for CD31 (CD31-PB), negatively for CD45.2 (CD45-APC-Cy7), and positively for Ki-67 (Ki-67-PE) were quantified. Results for cells stained with isotype controls for CD31 or Ki-67 in lieu of the respective antibody are shown in the inset. The percentage of cardiac endothelial cells (CD31+/CD45.2-) in each ventricle undergoing DNA replication (Ki67+) after 1 week of CH-PH is shown in (**b**.) (control *n* = 3/group; CH and SuCH n = 4/group). CH-PH caused a statistically significant increase in RV endothelial cell proliferation that was not significantly attenuated by SU5416. There was no CH-PH induced increase in LV endothelial cell proliferation after 1 week. *P*-values beneath the X-axis are for one-way ANOVA in each ventricle; *P*-value above individual bars denotes between group differences on Tukey’s multiple comparison testing. Endothelial cell specificity of CD31 antibody shown in Additional file 4: Figure S4. In (**c**), immunohistochemical staining confirms the presence of proliferating (PCNA +; arrowheads) cells in the RV of mice exposed to 1 week of CH-PH in the presence and absence of SU5416

**Fig. 5:**
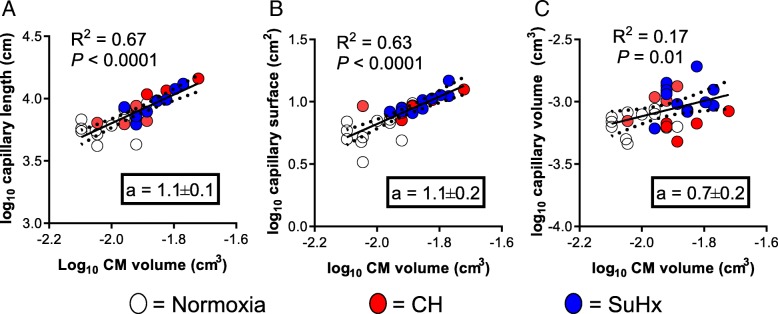
Effects of SU5416 on the relationship between RV microvascular remodeling and RV cardiomyocyte size. The relationship between total RV capillary length (**a**), surface area (**b**), and lumen volume (**c**) and RV cardiomyocyte (CM) volume is shown by linear regression. The slope of each line represents an estimate of relative growth, with a slope of 1.0 demonstrating isometry. Normoxic (open), CH-PH (red), and SU5416/CH-PH (blue) treated animals fall on the same curve. When compared against one another, the slopes were not significantly different (*P* = 0.60), suggesting similar rates of relative growth among all microvascular parameters (when compared with CM volume). Dotted lines represent 95% confidence interval of regression line

We subsequently used unbiased stereological methods to compare CH-PH induced increases in total RV capillary length, surface area, and volume between mice receiving SU5416 and vehicle at the one-week time point associated with increased VEGFR-2 expression. Surprisingly, SU5416 did not attenuate CH-PH induced increases in total RV capillary length, surface area, or volume (Fig. 3). In addition, SU5416 did not increase the radius of myocardial tissue served by each capillary (Fig. 3f), or significantly inhibit CH-PH induced RV EC proliferation (CD31+/CD45.2−/Ki-67+ cells; Fig. 4), though there was a non-significant reduction in RV EC proliferation in the SU5416 treated mice (*P* = 0.24 vs. CH-PH, Tukey’s multiple comparisons test). When the relationship between total RV capillary length or surface area and total RV cardiac myocyte volume was plotted, a strong association was consistently observed with a slope approximating one (Fig. 5), suggesting that capillary length (and surface area) increase proportionately with increases in myocyte hypertrophy. Total RV capillary volume (proportional to blood volume) was also associated with cardiac myocyte volume, though the relationship was not as robust. This parameter might be dependent on physiologic changes other than myocyte growth (e.g., adrenergic stimulation), and therefore more susceptible to variability at the time of tissue fixation, especially when immersion is used. Treatment with SU5416 did not alter the relationship between capillary length and myocyte hypertrophy, and there was no difference in slope of the regression lines generated for the separate CH and SuHx groups (*n* = 11/group, *P* = 0.52; not shown). Consistent with the lack of differences in RV angiogenesis between groups, there were no SU5416-mediated differences in CH-PH induced RV hypertrophy (Fulton index, RV/body weight) or RVSP 3 weeks after initiation of CH-PH (weekly SU5416 injections) (Table 1).

**Table 1:** Hemodynamics and RVH in murine CH-PH model

	Normoxia (*n* = 5)	CH-PH (n = 5)	Su/CH-PH (*n* = 7)	*P*-value
Weights				
Animal weight, g	28.2 ± 1.2	25.9 ± 0.8	25.4 ± 0.6	0.09
RV/BW, mg/g	0.68 ± 0.04	1.11 ± 0.08***	1.03 ± 0.05**	0.0004
Fulton’s index, RV/LV + S	0.229 ± 0.017	0.347 ± 0.018**	0.349 ± 0.015***	0.0003
LV + S/BW, mg/g	3.00 ± 0.12	3.21 ± 0.14	2.97 ± 0.15	0.48
Hemodynamics				
RVSP, mmHg	23.2 ± 1.6	28.2 ± 1.2*	28.9 ± 1.0*	0.01
RV d*P*/d*t*_max_, mmHg/s	1235 ± 178	1321 ± 123	1327 ± 70	0.84
RV d*P*/d*t*_min_, mmHg/s	− 1087 ± 135	− 1313 ± 115	− 1308 ± 110	0.36
HR, bpm	444 ± 21	435 ± 20	396 ± 12	0.12

*P*-value in Column 4 is for group ANOVA; Asterix reflect significance compared with Normoxia, at the given value: *P < 0.05, **P < 0.01, ***P < 0.001; RV = right ventricle; LV + S = left ventricle + septum; BW = body weight. Number of animals/group denoted in each column header

### SU5416 modifies RV angiogenesis gene expression in mice exposed to CH-PH

**Fig. 6:**
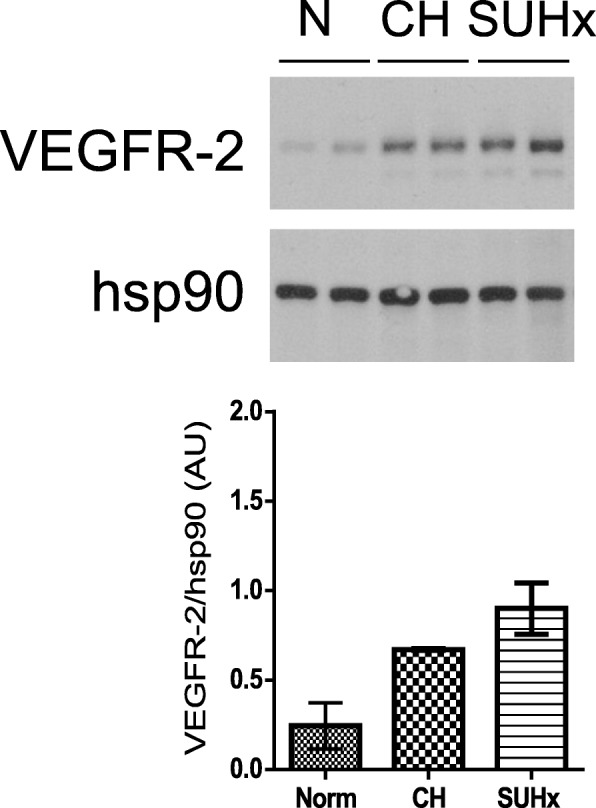
Effect of SU5416 on CH-PH induced RV VEGFR-2 expression. Western blot demonstrates that CH-PH induced increases in RV VEGFR-2 protein levels are not attenuated by SU5416. Hsp90 expression is shown as a reference for protein loading. Graph shows densitometry from this blot (*n* = 2/group). Array analysis similarly shows that CH-PH induced increases in *Kdr* transcription are not attenuated by SU5416 (Table 2)

Given the discordancy between our in vitro and in vivo observations, we hypothesized that activation of alternative and/or redundant pro-angiogenic pathways might support early angiogenesis in the presence of SU5416. Using a commercially available murine angiogenesis microarray (QIAGEN) containing 89 unique probes, we demonstrated increased RV expression (> 2-fold) of 29 genes after 1 week of CH-PH (Tables 2 and 3; full array results in Additional file 5 :Table S1). These included canonical pro-angiogenic growth factors and their receptors, cytokines, cell adhesion molecules, proteases, and ECM components. While 14 of these genes were also up-regulated by CH-PH in mice exposed to SU5416, CH-PH induced up-regulation of the remaining 15 genes was suppressed by SU5416 exposure. Concordant genes (Table 2) included growth factors/receptors, cytokine receptors, matrix components, and proteases. Among these, *Kdr* was upregulated in both groups, and western blot from RV tissue homogenates confirmed similar up-regulation of VEGFR-2 at the 1-week time point (Fig. 6). Discordant genes, suppressed by SU5416, included members of the VEGF-signaling pathway and Akt-1, a pro-angiogenic kinase activated downstream of VEGFR-2 via phospholipase C-gamma activation. Six genes were up-regulated by CH-PH exclusively in mice receiving SU5416. These included the inflammatory cytokines IL-6, IL-1β, and TNF-α. A single gene, *Angpt1* (encoding angiopoietin 1) was suppressed (compared to control) in the SU5416 treated CH-PH animals. Down-regulation of angiopoietin 1 is likely pro-angiogenic, as *Angpt1* deletion in adult mice has previously been associated with increased angiogenesis in a wound-healing model [32]. In total, there were 22 discordant genes between the CH-PH alone and CH-PH plus SU5416 groups (Table 3).

**Table 2:** Concordant Changes in Angiogenesis Profiler Array

Gene Identification		Fold-Change vs. Normoxia			
Symbol	Gene Name	CH-PH	*P*-value	SU/CH-PH	*P*-value
*Angpt2*	Angiopoietin 2	2.75	0.003788	2.61	0.005498
*Ccl2*	Chemokine (C-C) motif ligand 2	7.76	0.019500	8.39	0.024364
*Col18a1*	Collagen, type XVIII, alpha 1	3.70	0.000009	3.60	0.010498
*Cxcl2*	Chemokine (C-X-C) motif ligand 2	2.70	0.008983	3.24	0.005886
*Eng*	Endoglin	3.08	0.002967	2.02	0.034330
*Fn1*	Fibronectin 1	4.05	0.004777	3.19	0.031768
*Hgf*	Hepatocyte growth factor	2.85	0.000241	2.27	0.001051
*Kdr*	Kinase insert domain protein receptor	3.15	0.031063	2.08	0.049350
*Mmp14*	Matrix metallopeptidase 14	3.58	0.005553	2.77	0.002366
*Nrp2*	Neuropilin 2	3.50	0.004670	3.33	0.004227
*Pecam1*	Platelet/endothelial cell adhesion molecule-1	3.65	0.009939	2.33	0.049902
*Plau*	Plasminogen activator, urokinase	4.27	0.013232	3.07	0.014172
*Ptk2*	PTK2 protein tyrosine kinase 2	2.76	0.033783	2.03	0.012938
*Tgfb1*	Transforming growth factor, beta 1	2.77	0.011717	2.51	0.010149

*P*-values are for Student’s t-test comparing the experimental (n = 3/group) to control (Normoxia; n = 4) groups. CH-PH = chronic hypoxic pulmonary hypertension; SU/CH-PH = SU5416 + chronic hypoxic pulmonary hypertension

**Table 3:** Discordant Changes in Angiogenesis Profiler Array

Gene Identification		Fold-Change vs. Normoxia			
Symbol	Gene Name	CH-PH	*P*-value	SU/CH-PH	*P*-value
Transcripts Modified by CH-PH, but not SU/CH-PH					
*Akt1*	Thymoma viral proto-oncogene 1	2.22	0.007106	1.48	0.061133
*Cdh5*	Cadherin 5	3.65	0.000691	2.08	0.080106
*Efnb2*	Ephrin B2	2.29	0.042922	1.33	0.568661
*Ephb4*	Eph receptor B4	2.46	0.001461	1.94	0.007851
*Fgfr3*	Fibroblast growth factor receptor 3	2.01	0.047805	1.93	0.172211
*Flt1*	FMS-like tyrosine kinase 1	2.49	0.024092	1.51	0.033466
*Hif1a*	Hypoxia inducible factor 1, alpha subunit	2.42	0.012128	1.43	0.282336
*Nrp1*	Neuropilin 1	2.96	0.024777	1.80	0.166344
*Smad5*	MAD homolog 5 (Drosophila)	2.50	0.041301	1.43	0.224967
*Tgfb3*	Transforming growth factor, beta 3	2.35	0.046875	1.38	0.339163
*Tnfsf12*	Tumor necrosis factor (ligand) superfamily 12	2.31	0.014032	1.18	0.601718
*Vegfa*	Vascular endothelial growth factor A	2.30	0.048161	1.69	0.05109
Transcripts Modified by SU/CH-PH, but not CH-PH alone					
*Angpt1*	Angiopoietin 1	1.04	0.891137	−2.34	0.012238
*Cxcl5*	Chemokine (C-X-C motif) ligand 5	3.56	0.05293	2.43	0.031585
*Il1b*	Interleukin 1 beta	2.23	0.200162	2.29	0.034526
*Il6*	Interleukin 6	2.46	0.066759	2.60	0.022390
*Mmp19*	Matrix metallopeptidase 19	2.06	0.131679	2.87	0.002957
*Tgfbr1*	Transforming growth factor, beta receptor 1	2.71	0.082286	2.15	0.042879
*Tnf*	Tumor necrosis factor	3.15	0.070061	2.68	0.002550

*P*-values are for Student’s t-test comparing the experimental (n = 3/group) to control (Normoxia; n = 4) groups. CH-PH = chronic hypoxic pulmonary hypertension; SU/CH-PH = SU5416 + chronic hypoxic pulmonary hypertension

## Discussion

Animal models are important tools in our understanding of complex RV adaptive mechanisms in PH, including angiogenesis. Models intended to modify pulmonary vascular function may have unintended consequences for the RV. The VEGF-R inhibitor SU5416 is routinely employed in studies of RV failure, though its effects on cardiac EC function and RV angiogenesis is unknown. Given the broad phenotypic differences in ECs derived from different vascular beds [33], characterization of molecular regulators of proliferation and angiogenesis requires testing in ECs from the relevant vascular bed. Since we have recently demonstrated that RV EC proliferation and angiogenesis are induced during ventricular adaptation in the CH-PH model [6], and since others have proposed a direct effect of SU5416 on the RV in CH-PH [16], we hypothesized that the actively proliferating cardiac EC population might be a SU5416 target. We demonstrate here that SU5416 directly inhibited VEGF-mediated ERK phosphorylation, cell proliferation, and *Kdr* transcription, but not tube formation in primary murine cardiac ECs in vitro. However, the drug does not inhibit CH-PH induced RV angiogenesis, EC proliferation, or RV hypertrophy in vivo, despite significantly altering the RV expression profile of genes involved in angiogenesis. In sum, these findings demonstrate that SU5416 can directly alter isolated MCEC signaling pathways and myocardial expression of pro-angiogenic genes, but these effects are insufficient to functionally inhibit the more complex regulatory mechanisms controlling PH-induced myocardial angiogenesis in vivo. Further delineation of these mechanisms, in RV EC and other cell populations, will be necessary for development of future candidate therapeutic targets for RV angiogenesis.

Our results demonstrate that SU5416 alters MCEC function in ways that are consistent with those previously reported in other EC models. The competitive inhibitor of VEGF-R auto-phosphorylation and activation has been used to assess the role of VEGFR-2 tyrosine kinase activity in other EC models of proliferation and angiogenesis, primarily using primary human umbilical vein ECs (HUVEC). In our MCEC model, SU5416 inhibited VEGF-induced ERK phosphorylation and cell proliferation, but not tube formation on Matrigel. This is consistent with an inhibitory effect of SU5416 on early (proliferation) but not late (migration/differentiation) angiogenic function in MCECs. Previous studies have demonstrated that SU5416 prevents VEGF-mediated ERK phosphorylation and causes long-lasting inhibition of HUVEC proliferation [34] when administered at doses mimicking plasma levels in patients receiving the compound in clinical trials. Furthermore, SU5416 was previously shown to inhibit EC proliferation and sprouting, but not tube formation in an immortalized microvascular EC line derived from mouse heart (SMHEC4) [15]. Our current findings demonstrate a similar discordancy in primary cardiac ECs. We also observed direct inhibitory effects of SU5416 on MCEC transcriptional regulation of VEGFR-2 (*Kdr*), consistent with a previous report demonstrating that VEGFR-2 tyrosine kinase activity is necessary for VEGF-mediated transcriptional upregulation of *Kdr* in HUVECs [30]. Regulation of EC VEGFR-2 expression is complex, and includes both transcriptional and post-translational events that further regulate receptor activity [35].

Despite our in vitro findings, we observed no effects of SU5416 administration on RV angiogenesis or subsequent RV remodeling in the CH-PH model. In addition, the compound did not alter the linear relationship between capillary length and myocyte size (volume) in the model, suggesting that SU5416 does not inhibit matching of myocyte hypertrophy and myocardial angiogenesis during adaptive myocardial remodeling. The most notable difference between our in vitro and in vivo findings was the lack of inhibition of CH-PH induced RV EC proliferation by SU5416, despite robust inhibition of VEGF-stimulated MCEC proliferation in vitro. We suspect that this discrepancy reflects the increased complexity of mechanisms regulating angiogenesis and EC proliferation in vivo. For example, the pro-angiogenic effects of other growth factors or cytokines up-regulated in the RV by CH-PH in the presence of SU5416 (e.g., TGF-β, TNF-α; Tables 2 and 3) might mitigate effects of VEGFR-2 inhibition in vivo, but are not present in the in vitro model. Similarly, in vivo up-regulation of EC growth potentiators like fibronectin 1 [36] in the presence of SU5416 (Table 2) are not recapitulated in vitro. In addition, it is possible that the inhibitory effects of SU5416 on RV EC proliferation in vivo are “masked” by increased RV proliferation of other cell types not excluded by our flow cytometry gating protocol (e.g., CD31+ immune cells like macrophages), though our gating strategy excluded CD45 positive cells from analyses.

Our results do not exclude a role for VEGFR-2 tyrosine kinase activity during RV angiogenesis. To the contrary, we observed an RV-specific increase in VEGFR-2 expression (and *Kdr* transcription) in the mouse CH-PH model, which is temporally consistent with early RV EC proliferation and myocardial angiogenesis. In general, up-regulation of VEGFR-2 expression has previously been observed primarily during times of active EC proliferation in other models [9, 10]. We hypothesize that increased RV VEGFR-2 expression implies a critical role for the receptor in CH-PH induced RV angiogenesis. Previous assessment of RV VEGFR-2 expression in other PH models has been limited, though the findings support an association between VEGFR-2 and RV angiogenesis. In the SuHx rat model, *Kdr* transcript levels were decreased in late disease, and this was associated with reduced RV capillary density and RV failure [1]. Moreover, treatment with carvedilol was associated with increased *Kdr* transcript levels, increased RV capillary density, and improved cardiac function in the model [2]. However, the relevance of this association in human disease is less clear, as RV samples from PAH patients with decompensated heart failure showed no change in RV VEGFR-2 expression [5]. However, the study demonstrated an important role for SPRED-1 as a downstream inhibitor of VEGFR-2 target ERK in PH-induced capillary rarefaction [5]. Our study was not designed to test the hypothesis that VEGFR-2 is necessary for RV angiogenesis, and it is extremely unlikely that a single compound will be sufficient to address this question. Furthermore, although SU5416 has a relatively high specificity for VEGFR-2 inhibition (IC_50_ for auto-phosphorylation ~ 1 μM; [12]), it is also a potent inhibitor of other receptor tyrosine kinases, including c-Kit and RET [37, 38], and lacks the specificity to address questions about VEGFR-2. Future gain or loss of function studies will require development of a tissue-specific, inducible model for EC VEGFR-2 expression. In addition, further insight regarding the stimuli for and timing of receptor up-regulation and activation will improve experiments aimed at targeting the receptor, as regulation of EC VEGFR-2 expression and signaling is complex and dynamic [35].

Since we were unable to directly confirm inhibitory effects of SU5416 on RV EC function in vivo, it is possible that the observed lack of inhibition of RV angiogenesis and remodeling is due to failed delivery of the drug to cardiac ECs. However, doses of SU5416 administered in vivo were identical to those previously shown to cause persistent changes in Bax expression in the pulmonary circulation after 1 week of CH-PH [39]. The compound is highly lipophilic, and previous studies in HUVECs have demonstrated that transient exposure to SU5416 leads to prolonged inhibition of VEGFR-2 activation and EC proliferation due to intracellular accumulation of the tyrosine kinase inhibitor [34]. These previous observations support a prolonged effect of the drug in ECs after transient exposure, and we observed significant changes in CH-PH induced RV gene expression one week after SU5416 administration, consistent with a direct effect of the compound on the myocardium. While no previous studies have evaluated the mouse RV for direct effects of SU5416, a recent report describes ultrastructural evidence of myocardial EC injury just 3 days after administration of SU5416 in the Sprague-Dawley SuHx rat model, with subsequent reductions in RV *vegf* RNA expression and ERK phosphorylation [40]. Others have previously reported on insufficient [7] or disorganized [1] RV angiogenesis in the SuHx rat model, and it is possible that direct effects of SU5416 on the remodeling RV endothelium contribute to these observations.

We observed considerable differences in the expression profiles of CH-PH mice treated with or without SU5416. Since there were no differences in RV angiogenesis or remodeling between the two groups, both concordant and discordant gene expression profiles provide preliminary insight regarding potential regulatory pathways in RV angiogenesis. It is possible that genes up-regulated in both experimental conditions reflect candidates that are necessary for RV angiogenesis in response to CH-PH, but not dependent on VEGFR-2 signaling. These include candidate growth factors (angiopoietin-2, hepatocyte growth factor, transforming growth factor-β), chemokine receptors, and matrix components and proteases. Interestingly, VEGFR-2 (*Kdr*) and its co-receptor neuropilin-2 are up-regulated in both models, perhaps further reflecting the importance of the receptor to RV angiogenesis. We do not view this observation as discrepant with our in vitro model, based on VEGF stimulation, as the regulation of *Kdr* transcription is complex and the stimulus for up-regulation in vivo has not been determined. In addition to VEGF, other physiologically relevant stimuli like shear stress and hypoxia have previously been linked to VEGFR-2 expression [41, 42]. Genes that are up-regulated in CH-PH, but not in the presence of SU5416, may reflect target effects of the compound on myocardial cells and signaling pathways that are either unnecessary or redundant for RV angiogenesis in this model. Alternatively, these changes could be reflective of inhibition of other tyrosine kinases not necessary for RV angiogenesis. Angiogenesis related genes up-regulated by CH-PH only in the presence of SU5416 include multiple pro-angiogenic inflammatory mediators (IL-1β, IL-6, TNFα) that may suggest an alternative pathway(s) promoting microvascular remodeling in the presence of VEGFR-2 tyrosine kinase blockade. Potentially supporting this hypothesis, a recent comparison of SUHx-induced RV changes between different rats strains associated reduced inflammatory cell infiltration with decreased RV capillarization (and death) in Fischer rats when compared with Sprague-Dawley [43]. The specific role of myocardial inflammation in RV angiogenesis during PH requires further exploration.

There are limitations to this study. As stated previously, we were unable to directly confirm inhibition of RV VEGFR-2 activation by SU5416 in the model, so we are unable to comment on the necessity of this receptor in CH PH-induced early RV angiogenesis. Further studies will be necessary to address this question. In addition, while we used gold-standard methodology for unbiased assessment of increases in capillary length, we did not pursue additional ultrastructural or physiological measures to assess the role of SU5416 on the functionality of new RV microvasculature. We therefore cannot comment directly on the potential in vivo effects of SU5416 on other important components of RV angiogenesis beyond EC proliferation. Finally, our observations are limited to a single species and animal model, which is broadly viewed as a model of RV adaption to mild PH. As such, our findings are insufficient to make assumptions regarding the necessity of RV angiogenesis to human disease or RV failure.

## Conclusions

In sum, we show that SU5416 directly inhibited primary MCEC VEGFR-2 pro-angiogenic signaling and proliferation in vitro but does not inhibit Matrigel tube formation or attenuate CH-PH induced RV angiogenesis in the murine model. We present discordant RV microarray results that may suggest potential candidate regulators of RV angiogenesis in the model worthy of further exploration.

## Additional files

Additional file 1: **Figure S1.** C57 BL/6 mouse primary cardiac endothelial cells (Cell Biologics; Chicago, IL) were grown in the presence of Alexa594-conjugated acetylated low-density lipoprotein (Ac-LDL; 5 μg/mL) for 4 h to confirm endothelial identity. Qualitatively, nearly all cells demonstrated fluorescent staining consistent with Ac-LDL uptake after 4 h. (PDF 3665 kb)

Additional file 2: **Figure S2.** Western blot analysis of left ventricle (LV) and lung tissue homogenates from mice exposed to normoxia or CH-PH for 1 week shows either no change in VEGFR-2 expression (LV; *n* = 4/group) or a statistically significant decrease in VEGFR-2 expression (lung; *n* = 3/group). *P*-values are from Student’s t-test. (PDF 64 kb)

Additional file 3: **Figure S3.** Densities for stereology assessment. Capillary length density (L_cap/RV_; A), surface density (S_cap/RV_; B), lumen volume density (V_cap/RV_; C), and cardiomyocyte density (V_myo/RV_; D) for stereological assessments are shown. Densities are converted to total capillary length, surface area, volume, or total myocyte volume by multiplying by the reference (RV) volume, as described in the methods section. *P*-values are for one-way ANOVA. There were no statistically significant comparisons vs. control (Norm) in *post-hoc* analysis. (PDF 60 kb)

Additional file 4: **Figure S4.** Specificity of anti-mouse CD31 antibody used to identify RV cardiac endothelial cells for in vivo flow cytometry experiments. Primary C57 BL/6 mouse cardiac endothelial cells (MCEC) and cardiac fibroblasts (MCFb; Cell Biologics cat. #C57–6049) cultured to confluence in complete growth media were stained and analyzed by flow cytometry using the protocol and gating strategy described above. All stain = cells stained with live/dead, anti-CD31, anti-CD45.2 antibodies; FMO = fluorescence minus one; cells stained with live/dead and anti-CD45.2 antibodies alone; CD31-Iso = cells stained with live/dead, anti-CD45.2 antibody, and the isotype control for anti-CD31 antibody. (PDF 61 kb)

Additional file 5: **Table S1.** Angiogenesis Profiler Array Data. (DOCX 32 kb)

## Data Availability

The datasets used and/or analyzed during the current study are available from the corresponding author on reasonable request.

## Abbreviations

ANOVA: Analysis of variance
CH-PH: Chronic hypoxia-induced pulmonary hypertension
DMEM: Dulbecco’s modified Eagle’s medium
EC: Endothelial cell
ERK: Extracellular signal-regulated kinase
GAPDH: Glyceraldehyde 3-phosphate dehydrogenase
HRP: Horseradish peroxidase
Hsp90: Heat shock protein 90
HUVEC: Human umbilical vein endothelial cell
IB4: Isolectin B_4_
IL: Interleukin
IUR: Isotropic uniform random
Kdr: Kinase insert domain receptor
LV: Left ventricle
MCEC: Mouse cardiac endothelial cell
PAH: Pulmonary arterial hypertension
PH: Pulmonary hypertension
RV: Right ventricle
SDS-PAGE: Sodium dodecyl sulfate polyacrylamide gel electrophoresis
SuHx: SU5416 combined with chronic hypoxia exposure
TNF: Tumor necrosis factor
VEGF: Vascular endothelial cell growth factor
VEGFR-2: Vascular endothelial cell growth factor receptor-2
WGA: Wheat germ agglutinin
