# Supplementary figures and images for: SU5416 does not attenuate early RV angiogenesis in the murine chronic hypoxia PH model
Source: Respir Res. 2019 Jun 17;20:123. doi: 10.1186/s12931-019-1079-x (PMC6580559; doi:10.1186/s12931-019-1079-x)

Figure S1

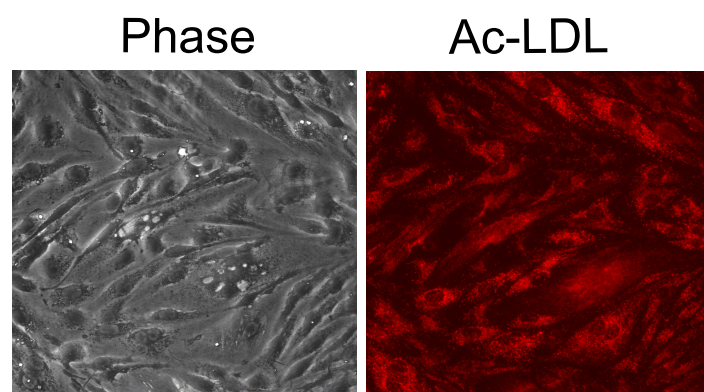

Supplement: Supplementary file 1 — Figure S1. C57 BL/6 mouse primary cardiac endothelial cells (Cell Biologics; Chicago, IL) were grown in the presence of Alexa594-conjugated acetylated low-density lipoprotein (Ac-LDL; 5 μg/mL) for 4 h to confirm endothelial identity. Qualitatively, nearly all cells demonstrated fluorescent staining consistent with Ac-LDL uptake after 4 h. (PDF 3665 kb) [file 12931_2019_1079_MOESM1_ESM.pdf]

Figure S2

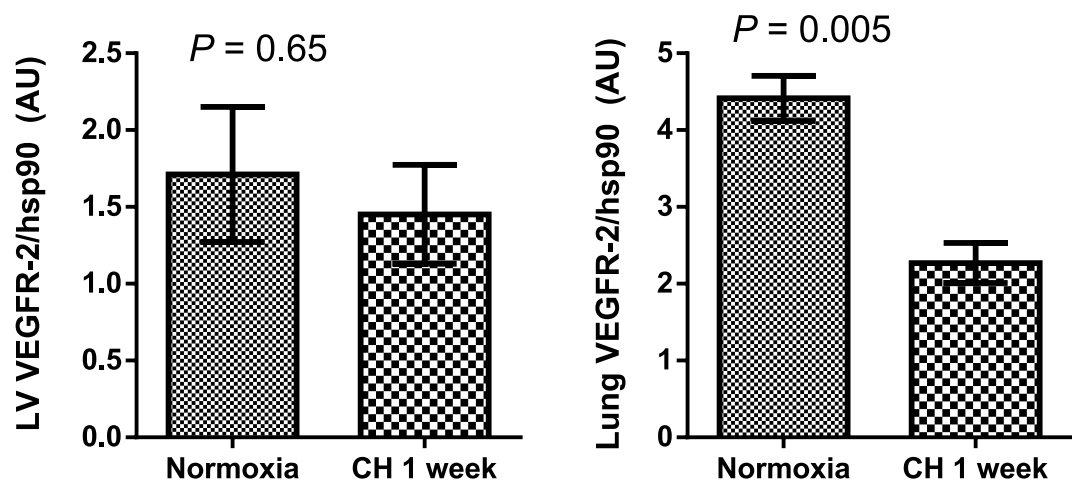

Supplement: Supplementary file 2 — Figure S2. Western blot analysis of left ventricle (LV) and lung tissue homogenates from mice exposed to normoxia or CH-PH for 1 week shows either no change in VEGFR-2 expression (LV; n = 4/group) or a statistically significant decrease in VEGFR-2 expression (lung; n = 3/group). P-values are from Student’s t-test. (PDF 64 kb) [file 12931_2019_1079_MOESM2_ESM.pdf]

Figure S3

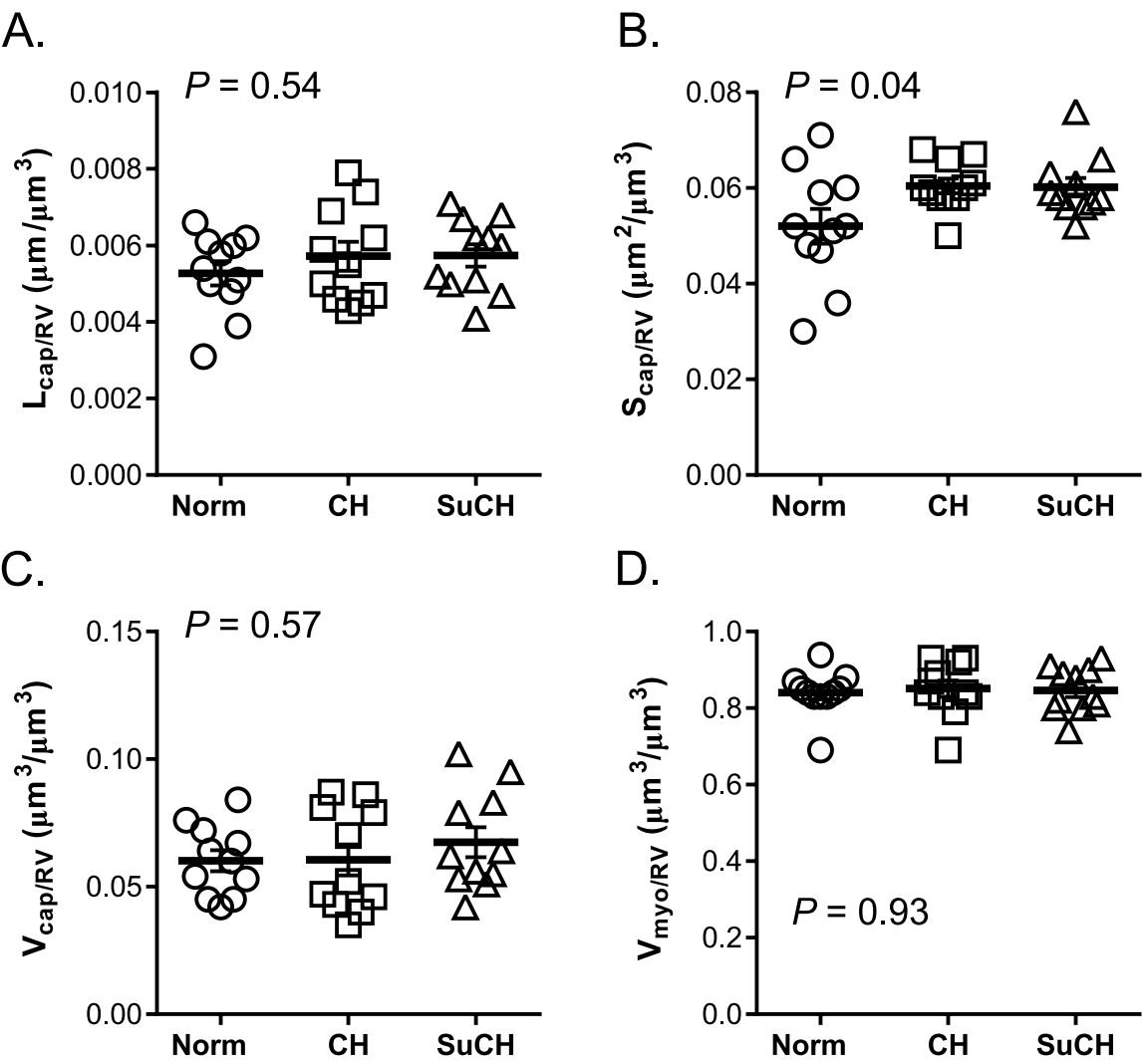

Supplement: Supplementary file 3 — Figure S3. Densities for stereology assessment. Capillary length density (Lcap/RV; A), surface density (Scap/RV; B), lumen volume density (Vcap/RV; C), and cardiomyocyte density (Vmyo/RV; D) for stereological assessments are shown. Densities are converted to total capillary length, surface area, volume, or total myocyte volume by multiplying by the reference (RV) volume, as described in the methods section. P-values are for one-way ANOVA. There were no statistically significant comparisons vs. control (Norm) in post-hoc analysis. (PDF 60 kb) [file 12931_2019_1079_MOESM3_ESM.pdf]

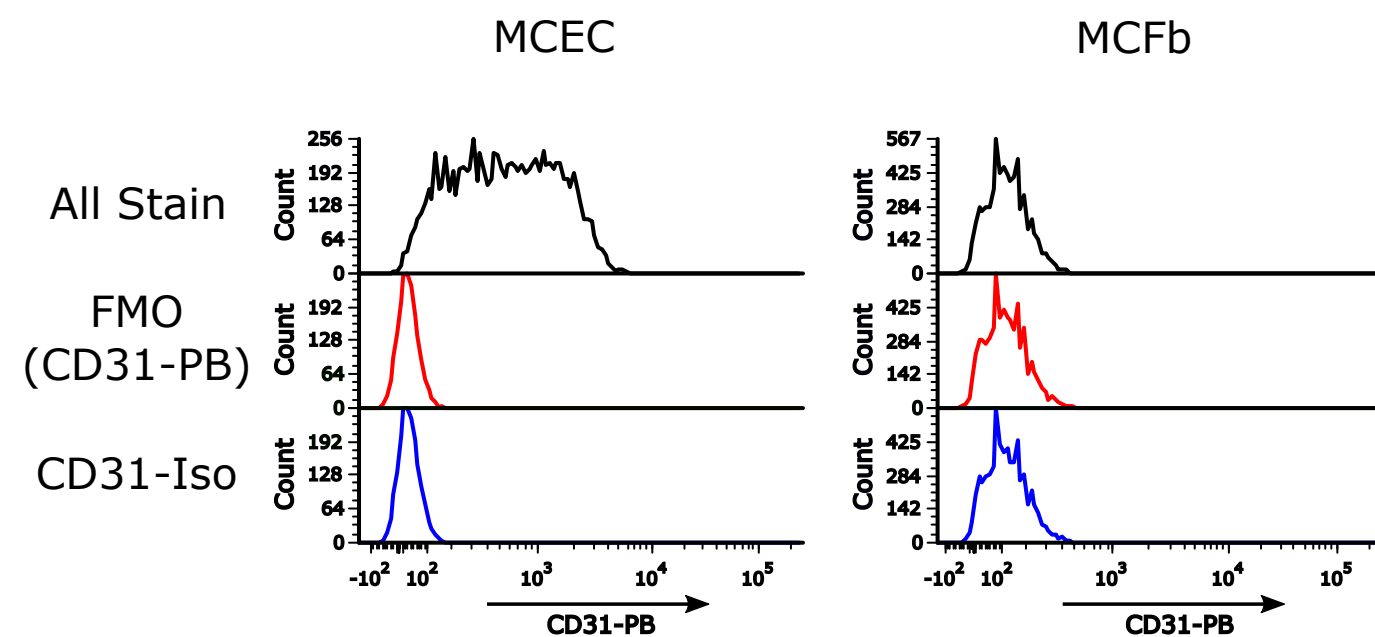

Supplement: Supplementary file 4 — Figure S4. Specificity of anti-mouse CD31 antibody used to identify RV cardiac endothelial cells for in vivo flow cytometry experiments. Primary C57 BL/6 mouse cardiac endothelial cells (MCEC) and cardiac fibroblasts (MCFb; Cell Biologics cat. #C57–6049) cultured to confluence in complete growth media were stained and analyzed by flow cytometry using the protocol and gating strategy described above. All stain = cells stained with live/dead, anti-CD31, anti-CD45.2 antibodies; FMO = fluorescence minus one; cells stained with live/dead and anti-CD45.2 antibodies alone; CD31-Iso = cells stained with live/dead, anti-CD45.2 antibody, and the isotype control for anti-CD31 antibody. (PDF 61 kb) [file 12931_2019_1079_MOESM4_ESM.pdf]
