# Supplementary material for: SU5416 does not attenuate early RV angiogenesis in the murine chronic hypoxia PH model
Source: Respir Res. 2019 Jun 17;20:123. doi: 10.1186/s12931-019-1079-x (PMC6580559; doi:10.1186/s12931-019-1079-x)
Supplement: Supplementary file 5 — Table S1. Angiogenesis Profiler Array Data. (DOCX 32 kb) [file 12931_2019_1079_MOESM5_ESM.docx]

| Gene Identification | | | Fold-Change vs. Normoxia | | | |
| --- | --- | --- | --- | --- | --- | --- |
| Symbol | Gene Name | RefSeq | CH-PH | *P*-value | SU/CH-PH | *P*-value |
| *Akt1* | Thymoma viral proto-oncogene 1 | NM_009652 | 2.22 | 0.007106 | 1.48 | 0.061133 |
| *Ang* | Angiogenin, ribonuclease, RNase A family, 5 | NM_007447 | 1.36 | 0.259691 | 1.41 | 0.172881 |
| *Angpt1* | Angiopoietin 1 | NM_009640 | 1.04 | 0.891137 | -2.34 | 0.012238 |
| *Angpt2* | Angiopoietin 2 | NM_007426 | 2.75 | 0.003788 | 2.61 | 0.005498 |
| *Anpep* | Alanyl (membrane) aminopeptidase | NM_008486 | 1.75 | 0.065664 | 1.19 | 0.585508 |
| *Adgrb1* | Brain-specific angiogenesis inhibitor 1 | NM_174991 | 1.36 | 0.259691 | 1.41 | 0.172881 |
| *Ccl11* | Chemokine (C-C motif) ligand 11 | NM_011330 | 1.06 | 0.874826 | 1.30 | 0.479919 |
| *Ccl2* | Chemokine (C-C motif) ligand 2 | NM_011333 | 7.76 | 0.0195 | 8.39 | 0.024364 |
| *Cdh5* | Cadherin 5 | NM_009868 | 3.65 | 0.000691 | 2.08 | 0.080106 |
| *Col18a1* | Collagen, type XVIII, alpha 1 | NM_009929 | 3.70 | 0.000009 | 3.60 | 0.010498 |
| *Col4a3* | Collagen, type IV, alpha 3 | NM_007734 | 2.54 | 0.09962 | 2.42 | 0.070074 |
| *Csf3* | Colony stimulating factor 3 (granulocyte) | NM_009971 | 1.36 | 0.259691 | 1.41 | 0.172881 |
| *Ctgf* | Connective tissue growth factor | NM_010217 | 2.27 | 0.151919 | 2.85 | 0.165863 |
| *Cxcl1* | Chemokine (C-X-C motif) ligand 1 | NM_008176 | 2.72 | 0.077159 | 1.42 | 0.21261 |
| *Cxcl2* | Chemokine (C-X-C motif) ligand 2 | NM_009140 | 2.70 | 0.008983 | 3.24 | 0.005886 |
| *Cxcl5* | Chemokine (C-X-C motif) ligand 5 | NM_009141 | 3.56 | 0.05293 | 2.43 | 0.031585 |
| *Edn1* | Endothelin 1 | NM_010104 | 2.18 | 0.062896 | 1.13 | 0.977888 |
| *Efna1* | Ephrin A1 | NM_010107 | 1.95 | 0.103156 | 1.21 | 0.782764 |
| *Efnb2* | Ephrin B2 | NM_010111 | 2.29 | 0.042922 | 1.33 | 0.568661 |
| *Egf* | Epidermal growth factor | NM_010113 | 1.00 | REF | 1.00 | REF |
| *Eng* | Endoglin | NM_007932 | 3.08 | 0.002967 | 2.02 | 0.03433 |
| *Epas1* | Endothelial PAS domain protein 1 | NM_010137 | 1.63 | 0.047784 | 1.03 | 0.748341 |
| *Ephb4* | Eph receptor B4 | NM_010144 | 2.46 | 0.001461 | 1.94 | 0.007851 |
| *Erbb2* | V-erb-b2 erythroblastic leukemia viral oncogene homolog 2, neuro/glioblastoma derived oncogene homolog (avian) | NM_001003817 | 1.92 | 0.011983 | 1.54 | 0.111974 |
| *F2* | Coagulation factor II | NM_010168 | 1.40 | 0.242328 | 1.64 | 0.023198 |
| *F3* | Coagulation factor III | NM_010171 | 1.51 | 0.25543 | -1.14 | 0.67547 |
| *Fgf1* | Fibroblast growth factor 1 | NM_010197 | 1.22 | 0.694445 | -1.64 | 0.334566 |
| *Fgf2* | Fibroblast growth factor 2 | NM_008006 | 1.60 | 0.373307 | 1.29 | 0.647125 |
| *Fgf6* | Fibroblast growth factor 6 | NM_010204 | 1.38 | 0.251017 | 1.41 | 0.172881 |
| *Fgfr3* | Fibroblast growth factor receptor 3 | NM_008010 | 2.01 | 0.047805 | 1.93 | 0.172211 |
| *Figf* | C-fos induced growth factor | NM_010216 | 1.25 | 0.746366 | 1.52 | 0.393621 |
| *Flt1* | FMS-like tyrosine kinase 1 | NM_010228 | 2.49 | 0.024092 | 1.51 | 0.033466 |
| *Fn1* | Fibronectin 1 | NM_010233 | 4.05 | 0.004777 | 3.19 | 0.031768 |
| *Hgf* | Hepatocyte growth factor | NM_010427 | 2.85 | 0.000241 | 2.27 | 0.001051 |
| *Hif1a* | Hypoxia inducible factor 1, alpha subunit | NM_001313919 | 2.42 | 0.012128 | 1.43 | 0.282336 |
| *Ifng* | Interferon gamma | NM_008337 | 1.36 | 0.259691 | 1.73 | 0.159481 |
| *Igf1* | Insulin-like growth factor 1 | NM_010512 | 2.48 | 0.056578 | 1.54 | 0.277603 |
| *Il1b* | Interleukin 1 beta | NM_008361 | 2.23 | 0.200162 | 2.29 | 0.034526 |
| *Il6* | Interleukin 6 | NM_001314054 | 2.46 | 0.066759 | 2.60 | 0.02239 |
| *Itgav* | Integrin alpha V | NM_008402 | 2.17 | 0.075314 | 1.53 | 0.178531 |
| *Itgb3* | Integrin beta 3 | NM_016780 | 1.99 | 0.100279 | 2.75 | 0.196295 |
| *Jag1* | Jagged 1 | NM_013822 | 2.00 | 0.114387 | 1.69 | 0.19694 |
| *Kdr* | Kinase insert domain protein receptor | NM_010612 | 3.15 | 0.031063 | 2.08 | 0.04935 |
| *Lect1* | Leukocyte cell derived chemotaxin 1 | NM_010701 | 1.48 | 0.227541 | 1.53 | 0.137566 |
| *Lep* | Leptin | NM_008493 | 1.36 | 0.259691 | 1.41 | 0.172881 |
| *Mapk14* | Mitogen-activated protein kinase 14 | NM_011951 | 1.60 | 0.083239 | 1.14 | 0.446565 |
| *Mdk* | Midkine | NM_010784 | 2.03 | 0.126874 | 1.88 | 0.219211 |
| *Mmp14* | Matrix metallopeptidase 14 (membrane-inserted) | NM_008608 | 3.58 | 0.005553 | 2.77 | 0.002366 |
| *Mmp19* | Matrix metallopeptidase 19 | NM_021412 | 2.06 | 0.131679 | 2.87 | 0.002957 |
| *Mmp2* | Matrix metallopeptidase 2 | NM_008610 | 2.42 | 0.067557 | 1.77 | 0.044811 |
| *Mmp9* | Matrix metallopeptidase 9 | NM_013599 | -1.00 | 0.853194 | 1.47 | 0.366646 |
| *Nos3* | Nitric oxide synthase 3, endothelial cell | NM_008713 | 2.05 | 0.090197 | 1.59 | 0.167251 |
| *Nrp1* | Neuropilin 1 | NM_008737 | 2.96 | 0.024777 | 1.80 | 0.166344 |
| *Nrp2* | Neuropilin 2 | NM_010939 | 3.50 | 0.00467 | 3.33 | 0.004227 |
| *Pdgfa* | Platelet derived growth factor, alpha | NM_008808 | 1.87 | 0.092733 | 1.31 | 0.345167 |
| *Pecam1* | Platelet/endothelial cell adhesion molecule 1 | NM_008816 | 3.65 | 0.009939 | 2.33 | 0.049902 |
| *Pgf* | Placental growth factor | NM_008827 | 1.77 | 0.00184 | 1.53 | 0.119105 |
| *Plau* | Plasminogen activator, urokinase | NM_008873 | 4.27 | 0.013232 | 3.07 | 0.014172 |
| *Plg* | Plasminogen | NM_008877 | 1.36 | 0.259691 | 1.41 | 0.172881 |
| *Ptgs1* | Prostaglandin-endoperoxide synthase 1 | NM_008969 | 1.93 | 0.054742 | 1.67 | 0.11785 |
| *Ptk2* | PTK2 protein tyrosine kinase 2 | NM_007982 | 2.76 | 0.033783 | 2.03 | 0.012938 |
| *S1pr1* | Sphingosine-1-phosphate receptor 1 | NM_007901 | 2.73 | 0.098409 | 1.47 | 0.474535 |
| *Serpine1* | Serine (or cysteine) peptidase inhibitor, clade E, member 1 | NM_008871 | -1.13 | 0.909254 | -1.16 | 0.57806 |
| *Serpinf1* | Serine (or cysteine) peptidase inhibitor, clade F, member 1 | NM_011340 | 2.33 | 0.113187 | 1.80 | 0.250414 |
| *Smad5* | MAD homolog 5 (Drosophila) | NM_008541 | 2.50 | 0.041301 | 1.44 | 0.224967 |
| *Sphk1* | Sphingosine kinase 1 | NM_025367 | 1.86 | 0.063162 | 1.68 | 0.021039 |
| *Tbx1* | T-box 1 | NM_011532 | 1.72 | 0.021911 | 1.37 | 0.124703 |
| *Tek* | Endothelial-specific receptor tyrosine kinase | NM_013690 | 2.20 | 0.084336 | 1.35 | 0.474614 |
| *Tgfa* | Transforming growth factor alpha | NM_031199 | 1.36 | 0.259691 | 1.41 | 0.172881 |
| *Tgfb1* | Transforming growth factor, beta 1 | NM_011577 | 2.77 | 0.011717 | 2.51 | 0.010149 |
| *Tgfb2* | Transforming growth factor, beta 2 | NM_009367 | 3.05 | 0.084247 | 1.93 | 0.372083 |
| *Tgfb3* | Transforming growth factor, beta 3 | NM_009368 | 2.35 | 0.046875 | 1.38 | 0.339163 |
| *Tgfbr1* | Transforming growth factor, beta receptor I | NM_009370 | 2.71 | 0.082286 | 2.15 | 0.042879 |
| *Thbs1* | Thrombospondin 1 | NM_011580 | 2.62 | 0.142265 | 4.11 | 0.061278 |
| *Thbs2* | Thrombospondin 2 | NM_011581 | 1.56 | 0.379454 | 1.02 | 0.998609 |
| *Tie1* | Tyrosine kinase with immunoglobulin-like and EGF-like domains 1 | NM_011587 | 2.13 | 0.053685 | 1.35 | 0.160504 |
| *Timp1* | Tissue inhibitor of metalloproteinase 1 | NM_011593 | 5.11 | 0.079095 | 5.74 | 0.051162 |
| *Timp2* | Tissue inhibitor of metalloproteinase 2 | NM_011594 | 2.18 | 0.12626 | 1.09 | 0.705906 |
| *Tnf* | Tumor necrosis factor | NM_013693 | 3.15 | 0.070061 | 2.68 | 0.00255 |
| *Tnfsf12* | Tumor necrosis factor (ligand) superfamily, member 12 | NM_011614 | 2.31 | 0.014032 | 1.18 | 0.601718 |
| *Tymp* | Thymidine phosphorylase | NM_138302 | 1.36 | 0.259691 | 1.45 | 0.126234 |
| *Vegfa* | Vascular endothelial growth factor A | NM_009505 | 2.30 | 0.048161 | 1.69 | 0.05109 |
| *Vegfb* | Vascular endothelial growth factor B | NM_011697 | 1.74 | 0.177006 | -1.41 | 0.299481 |
| *Vegfc* | Vascular endothelial growth factor C | NM_009506 | 1.56 | 0.387926 | 1.32 | 0.689531 |
| *Actb* | Actin, beta | NM_007393 | 4.42 | 0.021324 | 2.59 | 0.069436 |
| *B2m* | Beta-2 microglobulin | NM_009735 | 3.69 | 0.036629 | 2.88 | 0.070788 |
| *Gapdh* | Glyceraldehyde-3-phosphate dehydrogenase | NM_008084 | 3.18 | 0.121678 | 1.40 | 0.645662 |
| *Gusb* | Glucuronidase, beta | NM_010368 | 3.19 | 0.015893 | 2.11 | 0.080326 |
| *Hsp90ab1* | Heat shock protein 90 alpha (cytosolic), class B member 1 | NM_008302 | 1.88 | 0.15588 | 1.05 | 0.997778 |
